# Task-Irrelevant Context Learned Under Rapid Display Presentation: Selective Attention in Associative Blocking

**DOI:** 10.3389/fpsyg.2021.675848

**Published:** 2021-05-21

**Authors:** Xuelian Zang, Leonardo Assumpção, Jiao Wu, Xiaowei Xie, Artyom Zinchenko

**Affiliations:** ^1^Center for Cognition and Brain Disorders, Affiliated Hospital of Hangzhou Normal University, Hangzhou, China; ^2^Institute of Psychological Sciences, College of Education, Hangzhou Normal University, Hangzhou, China; ^3^Department Psychologie, Ludwig-Maximilians-Universität München, Munich, Germany

**Keywords:** contextual cueing, rapid presentation, contextual learning, attention, associative blocking, task-irrelevant context

## Abstract

In the contextual cueing task, visual search is faster for targets embedded in invariant displays compared to targets found in variant displays. However, it has been repeatedly shown that participants do not learn repeated contexts when these are irrelevant to the task. One potential explanation lays in the idea of *associative blocking*, where salient cues (task-relevant old items) block the learning of invariant associations in the task-irrelevant subset of items. An alternative explanation is that the associative blocking rather hinders the allocation of attention to task-irrelevant subsets, but not the learning *per se*. The current work examined these two explanations. In two experiments, participants performed a visual search task under a rapid presentation condition (300 ms) in Experiment 1, or under a longer presentation condition (2,500 ms) in Experiment 2. In both experiments, the search items within both old and new displays were presented in two colors which defined the irrelevant and task-relevant items within each display. The participants were asked to search for the target in the relevant subset in the learning phase. In the transfer phase, the instructions were reversed and task-irrelevant items became task-relevant (and vice versa). In line with previous studies, the search of task-irrelevant subsets resulted in no cueing effect post-transfer in the longer presentation condition; however, a reliable cueing effect was generated by task-irrelevant subsets learned under the rapid presentation. These results demonstrate that under rapid display presentation, global attentional selection leads to global context learning. However, under a longer display presentation, global attention is blocked, leading to the exclusive learning of invariant relevant items in the learning session.

## Introduction

**Figure 1:**
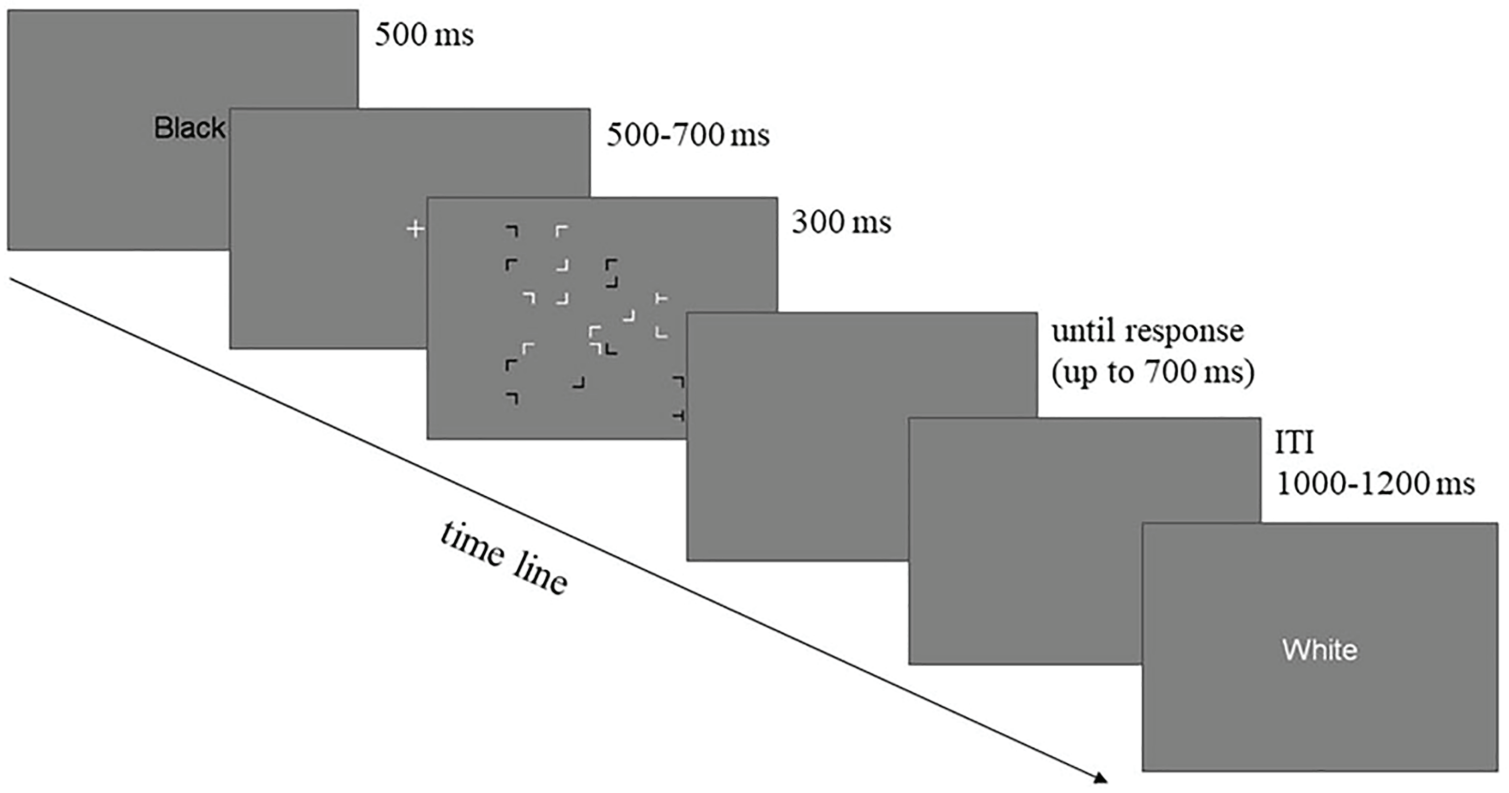
Schematic illustration of the trial sequence in Experiment 1. A word cue (“Black” or “White”) was presented first, indicating the target color of the trial. After a 500–700 ms central fixation, the search display was rapidly presented for 300 ms, followed by a 700 ms blank screen both of which participants could use to produce the response (total of 1,000 ms). Lastly, the inter-trial interval was presented for 1,000–1,200 ms when the response window closed.

Our visual system evolved to take advantage of spatial regularities in the environment to facilitate visual search. Objects in our surroundings do not appear at random locations every time they are encountered; instead, they tend to be represented in a quasi-ordered fashion, thus forming spatial regularities, also referred to as spatial contexts. For example, items on supermarket aisles and shelves are likely to be placed in a very similar arrangement across different supermarkets, thus making the supermarket context so similar that shoppers have no difficulty finding their products even though they might be shopping in a different neighborhood. In the lab setting, the context-guided performance was first demonstrated by Chun and Jiang (1998) who used an elegant visual search task to investigate how repeated configurations of items (contexts) could facilitate search performance. In detail, the authors asked participants to search and identify the tilt (left, right tilt; see Figure 1 for an example) of the target letter “T” that was surrounded by task-irrelevant letters “L.” Unknown to participants, the authors repeated target-distractor spatial contexts in 50% of all search trials. It was shown that, over time, participants’ search time became faster for repeated contexts compared to the search of random contexts, a phenomenon termed as contextual cueing effect. The idea behind this finding is that repeated contexts are learned, consequently orienting participants’ attention to the target (Chun and Jiang, 1998; Sisk et al., 2019). Simply put, learned spatial contexts facilitate attentional processes and improve visual search.

Interestingly, the extensive investigation of the contextual cueing effect in the past decades has shown a bidirectional interaction between repeated contexts and visual attention. Meaning that in addition to the classic attentional guidance account cited above, the availability of attentional resources can further modulate the acquisition of invariant spatial configurations (e.g., Jiang and Chun, 2001; Geyer et al., 2010; but see Jiang and Leung, 2005). This was first demonstrated by Jiang and Chun (2001, Experiment 3), where participants searched for a colored (red and green) target item that was presented among equal subsets of green and red distractors. The participants had to attend to only one color-defined subset of items while ignoring the rest of the irrelevant-color items. In total, the authors introduced four context conditions: (i) the control condition, where locations of target and distractors in both color sets were chosen randomly on each trial, (ii) attended-old condition, where locations of target and distractor items were fixed only in the to-be-attended color set, but not in the to-be-ignored set, (iii) ignored-old condition, where contexts were invariant in the to-be-ignored color set, and finally, (iv) both-old condition, where search displays were repeated in both color sets. The results showed significant contextual cueing effects for both the both-old and attended-old conditions. For the ignored-old condition, only one of four experiments (i.e., Experiment 3) revealed a modest contextual cueing effect of 31 ms. Since invariant, but ignored configurations did not result in a reliable cueing effect, the authors concluded that robust and stable target-distractor associations require selective attention.

These findings were further corroborated and extended by Jiang and Leung (2005) who used a similar design to that of Jiang and Chun (2001) regarding the training session. For instance, participants searched for a target letter that was presented within one of the two target colors (black and white) under four search conditions, namely, both-old, both-new, attended-old, and attended-new. Additionally, the authors introduced a transfer phase in which the instructions regarding the color to attend were reversed. For example, item subsets that were ignored in the preceding training session became task-relevant in the transfer session, whereas previously attended items in the training session became task-irrelevant. As a result, in the learning phase, the visual search was speeded in conditions where a target item was paired with both repeated and task-relevant sets of items (both-old and attended-old conditions). By contrast, search performance was not improved when the repeated items were presented in the task-irrelevant color in the ignore-old condition. Interestingly, in the transfer phase, a significant contextual cueing effect was observed immediately for the (previously) ignored-old but not for both-old and attended-old conditions. Jiang and Leung (2005) concluded that the expression of learned contextual regularities requires selective attention, while the build-up of these memories (i.e., contextual learning) does not. However, this idea was further developed by Vadillo et al. (2020) when they reevaluated the role of selective attention in contextual cueing. The study employed a similar design to Jiang and Leung (2005) with a larger sample size and showed that no latent learning would be possible without selective attention. The authors then demonstrated that selective attention is equally important for both the acquisition and expression of contextual information.

To explain the lack of contextual cueing in their study, Jiang and Leung (2005) proposed a mechanism of associative blocking in contextual cueing. Associative blocking was first demonstrated by Kamin (1969) who showed that the presence and association of a salient cue and the target can block an association between a less salient cue and the target. Applied to contextual cueing, it means that the task-relevant repeated contexts (salient cues) weaken the association between the target and task-irrelevant repeated contexts (less salient cue; see also Endo and Takeda, 2004 and Geyer et al., 2021), thus hindering the acquisition of task-irrelevant context in the both-old condition. By contrast, when the salient cue was not present in the ignore-old condition, the association between task-irrelevant invariant context and the target was built and expressed in the transfer phase (i.e., there was no salient cue to block learning in terms of Kamin, 1969; Jiang and Leung, 2005). Although the authors highlighted the importance of selective attention in contextual cueing tasks, they did not answer whether associative blocking operates on the selective attention process (attentional blocking), or the cognitive processes following selective attention (e.g., the memory encoding and storage). For instance, attentional blocking may operate at the early stages of visual search, such as by color-segmentation. In detail, it has been suggested that participants can segment search items into task-relevant and -irrelevant based on the color information early in visual search and before the attentional selection of search items (Conci and Müller, 2014). Thus, while color segmentation may aid the rapid attentional selection of task-relevant items, it helps to filter out irrelevant search items (Conci and Müller, 2014; Zang et al., 2016, 2017). Subsequently, invariant subsets of items of task-irrelevant colors would not affect search performance in the transfer phase. The current study set out to investigate the nature of the relationship between attentional selection and associative blocking in the presence of a variety of contexts in the contextual cueing task, and how this relationship affects the learning and expression of the cueing effect. Precisely, we examined whether the previously observed post-transfer reduction in the cueing effect was primarily due to the lack/blocking of selective attention to the irrelevant subsets. To answer this question, we combined relevant-irrelevant color subsets in the learning and transfer phases with a rapid presentation paradigm to direct participants’ attention to the task-irrelevant subsets of items. In greater detail, in a recent study, Zang et al. (2020) demonstrated that participants were able to show a stable contextual cueing effect even when the search items were presented for only 300 ms. The authors revealed that the contextual cueing effect was established under rapid presentation only when the global configuration of items was repeated across trials. In contrast, no contextual cueing was formed under a 300 ms presentation when only a subset of items was repeated, such as when the repeated distractors were limited to the target quadrant, while distractors in the other quadrants varied randomly. Interestingly, with a similar manipulation of search items but under longer presentation time (i.e., displays presented until participants’ response or a maximum presentation duration of 8 s), Brady and Chun (2007) reported that participants could learn the local context within the target quadrant and engender the contextual cueing effect. Together, these results show that longer presentation time facilitates local context learning; however, the rapid display presentation only allows sufficient time for global attention. In other words, it forces participants’ attention to the whole global context relative to only a subset of items (see Zang et al., 2020).

Based on the aforementioned literature, we hypothesized that if the post-transfer reduction in contextual cueing is the result of lack/blocking of the attentional selection of the irrelevant subset of items, forcing participants’ attention to the whole display utilizing rapid presentation of search items (300 ms) in Experiment 1 should result in contextual cueing effect also in the transfer session. Particularly, the task-irrelevant context would also be acquired in the training session. In contrast, if selective attention is not the source of learning of task-irrelevant context, then forcing participants to concentrate on the whole context using rapid presentation would not result in any cueing effect post-transfer. This is because associative blocking would still hinder the learning of irrelevant context regularities in the original learning session. In Experiment 2, participants performed the contextual cueing task with display presentations of 2.5 s. This part of the study served as a control experiment with no specific manipulation of selective attention to the task-irrelevant context.

## Experiment 1

The purpose of Experiment 1 was to test whether the rapid presentation of search displays can improve contextual cueing for items previously presented in task-irrelevant color in the initial learning session. With this aim, each search display was presented for only 300 ms in the current experiment.

### Method

#### Participants

Twenty naive volunteers from Hangzhou Normal University (15 females; age range: 18–22 years) took part in Experiment 1 in exchange for monetary compensation. All of them reported normal or corrected-to-normal visual acuity. Before the start of the experiment, participants gave written informed consent. The sample size was calculated based on the samples and effect sizes reported in previous studies that included 8–20 participants (e.g., Geringswald et al., 2015; Zang et al., 2015; Zinchenko et al., 2018). Based on effect size measures provided in previous studies, our sample size is appropriate to detect an *f*(U) effect size of 0.57 with 95% power (*η*_p_^2^ = 0.25, groups = 2, number of measurements = 9), given an alpha level of 0.05 and a nonsphericity correction of 1. The current study was approved by the ethics committee of the Institutes of Psychological Sciences in Hangzhou Normal University.

#### Apparatus and Stimuli

The experiment was performed in a quiet, dim cabin (0.69 cd/m^2^). All stimuli were presented on a monitor (22-inch, 120 Hz) set in front of a fixed chin rest, with a viewing distance of 57 cm. Matlab (Mathworks, Natick, MA, United States) programs were used to present stimuli and record responses with the help of the Psychtoolbox extension (Brainard, 1997; Pelli, 1997). Each search display consisted of 20 items (1.0° × 1.0° in visual angle) presented on a gray background (RGB value = [80 80 80], luminance = 11.3 cd/m^2^). The displays had two subsets, each containing 10 items: the black subset (RGB value = [0 0 0], luminance = 0.7 cd/m^2^) and the white subset (RGB value = [255 255 255], luminance = 149.0 cd/m^2^). Note, the luminance contrasts of item-background [calculated as the Michelson contrast, (L_i_ − L_b_)/(L_i_ + L_b_), with L_i_ and L_b_ indicating the luminance of items and background, respectively, see Zang et al., 2020 for a similar approach] were comparable between the black and white items (0.88 and 0.86, respectively). In each subset, there was a “T”-shaped target and nine “L”-shaped distractors. In the displays, distractors were presented randomly in one of four orthogonal rotations (0, 90, 180, and 270°), while the targets were rotated either 90 or 270° clockwise. The two subsets of items were placed in an invisible 12-by-12 square grid, with one subset (the black or the white subset) randomly arranged in odd columns and the other in even columns. The “T”-shaped targets were uniformly distributed in the cells except for the center four and the corner four cells in the grid. The allocation and color in two subsets were randomly paired (black in odd columns and white in evens, or black in even columns and white in odds) and balanced between old and new configurations.

#### Design and Procedure

The experiment was composed of three sessions: a 50-block learning session, a 10-block transfer session, and an extra one-block recognition session. In the learning and the transfer sessions, each block contained 24 trials, with 12 old (i.e., repeated configurations) and 12 new (i.e., random configurations) trials being randomly and intermixedly presented. In other words, each old and new configuration was presented once per learning/transfer block, leading to 50 repetitions of the old configurations in the learning session and 10 repetitions in the transfer session. Each display had two targets (one black and one white) and 18 distractors (nine for each color). Participants were instructed to respond to only one target, with a word cue of the target color “Black” or “White” presented at the beginning of each trial. The target-relevant color was balanced within each block, which means that participants should respond to the white target in half of the trials and the black target in the other half of the trials. For each of the old displays, the location and orientation of distractors in both the task-relevant group and the task-irrelevant group, together with the location of both targets, were kept constant and repeated once per block. For each of the new displays, the location and orientation of distractors in both groups varied randomly. Note that the target location for each of the old and new configurations stayed constant during the whole experiment, rendering overall 48 target locations of which 24 were used for old display configurations and 24 for new in the entire experiment. In consideration of a potential confounding impact of response learning, the target orientation appeared randomly to the left or the right and was balanced across the whole experiment (for both old and new configurations).

In the learning session, each trial started with a 500 millisecond (ms) word cue (“Black” or “White”) informing participants of the target-relevant color of the trial (see Figure 1). The word “White” instructed participants to search for the white target, while the word “Black” indicated a search for the black target. Thereafter, a fixation cross was presented in the center of the screen for 500–700 ms randomly, followed by a search display consisting of “T”s and “L”s presented for 300 ms. The participants were instructed to search for the target and distinguish its direction as soon and as accurately as possible by pressing the corresponding response key (the left or right arrow key). Following the search display, a blank screen was presented for another 700 ms, during which participants could still make a response (the overall duration for making a response was 1,000 ms). After the response, an inter-trial interval (ITI) of 1,000–1,200 ms was presented before the start of the next trial.

The trial sequence in the transfer session was virtually the same as in the learning session, with the exception that the search display was presented until a response was produced or for no longer than 2,500 ms. Note that, the prolonged time window for the response was set in order to increase the external validity of the study, which means that the observed result could be directly extended to and compared with other contextual cueing studies with long presentation time (e.g., Brady and Chun, 2007). After the display presentation, the participants could still make a response during the first 500 ms presentation of a blank screen (maximum response window of 3,000 ms). Importantly, in half of the trials (consisting of randomly selected six old and six new displays), namely “reverse” trials, participants were instructed to search for a different colored target as compared to the learning session. For instance, if participants were instructed to search for the “Black” target in the initial learning session in a particular display, but now in the transfer session, participants were instructed to search for the “White” target. In other words, the display/configuration stays the same, but participants were instructed to search for a different target, while the color for the whole search configuration remained constant. That is, the color, location, and orientation of the stimuli under the reverse condition in the transfer session were identical to those used during the learning session; however, the instruction regarding the target color to search for was reversed. Thus, the original task-irrelevant stimuli (the items with a different color from the main target) in the learning phase became task-relevant in the transfer phase. By contrast, the other half of the trials, namely “original” trials kept the same search setting as that used in the learning session. This methodological approach allowed us to assess whether the magnitude of this target-irrelevant contextual learning effect was comparable to or relatively smaller than that induced by the target-relevant context.

Finally, the one-block recognition session was composed of trials with the 12 old configurations in the learning session and 12 newly generated random configurations. Participants were instructed to respond whether a given configuration had been presented in the learning session or not. The display remained on the screen until a response was produced, or for a maximum of 10 s. Thereafter, a blank ITI between 1,000 and 1,200 ms was presented. Prior to the experiment, participants performed a two-block practice session (each consisted of 12 old and 12 new trials). During practice, the displays were presented for 2,500 ms in the first block and 300 ms in the second block to gradually increase the task difficulty. None of the practice configurations were used in the main experiment.

To summarize, the current experiment applied a variant of the task-relevant contextual cueing paradigm, with the visual search displays being rapidly presented (only 300 ms). For each display, half of the search items were presented in white and the other half in black. The items containing the same color as the search target were deemed as task-relevant context while the other items were deemed as the task-irrelevant context. Importantly, the task-relevant and -irrelevant context reversed from the learning to the transfer session to examine whether the task-irrelevant context was also learned in the learning session. Therefore, if the initial contextual learning effect could not be transferred to the reversed display, we can conclude that the initial contextual cueing effect was established solely based on the learning of the task-relevant context. By contrast, if a significant contextual cueing effect (if any) is observed in the transfer session, we can infer that the task-irrelevant context was also learned in the previous learning session.

#### Statistical Analysis

To improve the power of statistical analysis, every five blocks were binned into one epoch, resulting in 10 and two epochs in the learning and transfer sessions, respectively. Trials with no or wrong responses were treated as error trials and were not included in the response time (RT) analysis. Greenhouse-Geisser correction was used when the sphericity of Mauchly’s test was violated. In addition, for the main effects that were not significant, the Bayes factors could further test the reliability of the null hypothesis results. We used JASP (version 0.9.1) to calculate the Bayes factor (Marsman and Wagenmakers, 2017), where the default Cauchy settings (i.e., *r*-scale fixed effects = 0.5, *r*-scale random effects = 1, and *r*-scale covariates = 0.354) and Cauchy prior (scale = 0.707) were used in ANOVA and *t*-test. *BF*_10_ represents the degree to which the data support the alternative hypothesis (i.e., H_1_) compared with the null hypothesis (H_0_). A *BF*_10_ value larger than 3 is considered to support the alternative hypothesis, otherwise, a positive *BF*_10_ value less than 1/3 is the evidence to support the null hypothesis (Wetzels et al., 2011).

### Results

#### Errors

**Figure 2:**
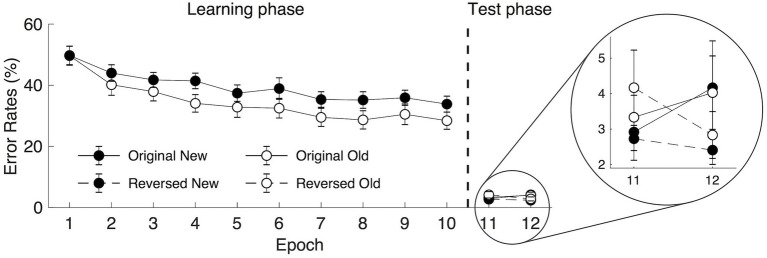
Mean error rates in the learning (epoch 1–10) and the transfer (epoch 11–12) phases as a function of epoch, context, and display type under rapid search display presentation for 300 ms. The error bars represent the within-subject SE of the mean. The black circle lines indicate the new context and the open circle lines indicate the old context. The solid line indicates the original context and the dashed line represents the reversed context.

Due to the limited presentation time of the visual displays (i.e., 300 ms), participants showed relatively high mean error rates, including trials with wrong and no responses (*M* = 36.89%) in the learning phase (see Figure 2). A repeated-measure ANOVA of the mean error rates with Context (old vs. new) and Epoch (1–10) as factors revealed significant main effects of both Context and Epoch: Context, *F*(1, 19) = 7.568, *p* = 0.013, *η*_p_^2^ = 0.285, with lower error rates for the old (34.43%) than for the new context (39.35%), mean difference = 4.92%; Epoch, *F*(3.535, 67.171) = 17.549, *p* < 0.001, *η*_p_^2^ = 0.480, with a decreased mean error rate of 18.58% from Epoch 1 (49.71%) to Epoch 10 (31.13%). The Context × Epoch interaction was not significant, *F*(9, 171) = 1.610, *p* = 0.116, *η*_p_^2^ = 0.078. When further analyzing the error trials with no response or wrong responses separately, we found significant main effects of Context and Epoch for the no response (i.e., miss trials) but not for the wrong response trials. For no response trials (mean of 21.96%): Context, *F*(1, 19) = 7.281, *p* = 0.014, *η*_p_^2^ = 0.277, with 23.53 and 20.38% miss rates of the new and old displays, respectively; Epoch, *F*(2.041, 38.778) = 9.939, *p* < 0.001, *η*_p_^2^ = 0.343, with a decreased mean miss rate of 19.96% from Epoch 1 (36.00%) to Epoch 10 (16.04%). The Context × Epoch interaction was not significant, *F*(9, 171) = 0.879, *p* = 0.545, *η*_p_^2^ = 0.044. For the wrong response trials (mean of 14.93%): Context, *F*(1, 19) = 3.528, *p* = 0.076, *η*_p_^2^ = 0.157, *BF*_10_ = 3.919, with mean wrong rates of 15.82 and 14.04% for the new and old contexts, respectively, and the *BF*_10_ value indicating that the probability of the alternative hypothesis being true is 3.92 times that of the null hypothesis; Epoch, *F*(2.429, 46.142) = 0.666, *p* = 0.547, *η*_p_^2^ = 0.034, *BF*_10_ = 0.054. The Context × Epoch interaction was not significant, *F*(9, 171) = 0.864, *p* = 0.559, *η*_p_^2^ = 0.044. Taken together, the results on the errors suggested that participants improved their search performance, including reducing the miss rates and wrong rates with the learning of the old context (i.e., contextual cueing effect) and the practice of the task (i.e., procedural learning).

In the following transfer session, the visual display was presented for a longer time (2,500 ms), resulting in a very low error rate (*M* = 3.12%, see Figure 2). Due to the lack of statistical power, the error rates in the transfer session were not included in the statistical analysis.

#### Reaction Times

##### Learning Phase

**Figure 3:**
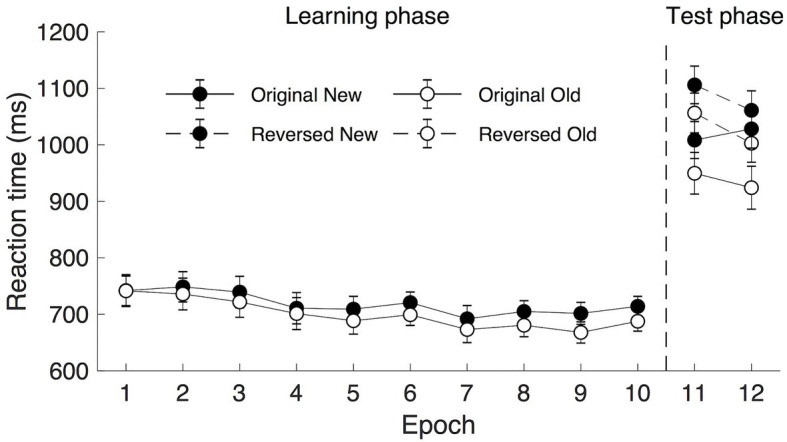
Mean response time (RT) as a function of epoch and context for rapid display presentation (300 ms). The error bars represent the within-subject SE of the mean. The black circle lines indicate the new context and the open circle lines indicate the old context. The solid line indicates the original context and the dashed line represents the reversed context.

The mean RTs are shown in Figure 3 (Epoch 1–10). A repeated measure ANOVA of RTs in the learning phase (under 300 ms display presentation) with factors Context (old vs. new) and Epoch (1–10) showed a significant main effect of Context [*F*(1, 19) = 9.265, *p* = 0.007, *η*_p_^2^ = 0.328, mean of 699 ms and 718 ms for old and new context, respectively], and a non-significant effect of Epoch [*F*(1.589, 30.182) = 2.674, *p* = 0.096, *η*_p_^2^ = 0.123, *BF*_10_ > 10]. The high *BF*_10_ value implicated a more than 10 times higher probability of the alternative hypothesis being true than that of the null hypothesis. Therefore, although the value of *p* did not reach significance, the *BF*_10_ value still suggested the occurrence of the procedural learning effect. The mean RTs were 741, 742, 731, 706, 699, 710, 683, 693, 685, and 701 ms of the 10 learning epochs, respectively. The interaction between Context and Epoch was significant, *F*(9, 171) = 2.065, *p* = 0.035, *η*_p_^2^ = 0.098. *Post hoc* analysis showed that there was a significant difference between old and new contexts from Epoch 5 (all *t*s > 2.139; all *p*s < 0.046, Cohen’s *d*s > 0.478, mean contextual cueing > 18 ms), suggesting that contextual cueing effect developed with the progress of the experiment.

##### Transfer Phase

A repeated measure ANOVA with factors Display Type (reverse vs. original), Context (old, new), and Epoch (11–12) were applied. For the Epoch, mean RTs were comparable in the two transfer epochs, *F*(1, 19) = 1.489, *p* = 0.237, *η*_p_^2^ = 0.073, *BF*_10_ = 0.408, mean of 1,030 and 1,004 ms, respectively. For the Context, a significant contextual cueing effect was observed (see Figure 3), participants’ response was faster to old displays (983 ms) than to the new displays (1,051 ms), *F*(1, 19) = 12.958, *p* = 0.002, *η*_p_^2^ = 0.405. For the Display Type, a significant main effect was observed, *F*(1, 19) = 13.910, *p* = 0.001, *η*_p_^2^ = 0.423, the mean RTs were 978 and 1,057 ms for the original and reversed displays, respectively, suggesting that searching for the alternative target (previously task-irrelevant set) in the transfer session engendered the overall RT cost, as participants became slower in searching for the target. Note the Display Type × Epoch interaction was also significant [*F*(1, 19) = 5.532, *p* = 0.030, *η*_p_^2^ = 0.226], mainly caused by larger mean RT differences between the original and the reversed display types in Epoch 11 (102 ms) than that in Epoch 12 (56 ms), *t*(19) = 2.352, *p* = 0.030, Cohen’s *d* = 0.526. No other interactions were significant [all *F*s < 2.88, all *p*s > 0.10]. Importantly, the lack of interaction between Display Type and Context demonstrated that the expression of the contextual cueing effect was comparable across the original and reversed display types (81 and 54 ms, respectively), demonstrating that participants learned the task-irrelevant context in the learning session.

To further examine contextual cueing transfer effect without potential influences of procedural learning, a repeated measure ANOVA of mean RTs in the first transfer epoch (i.e., Epoch 11) with factors Display Type (reverse vs. original) and Context (old vs. new) was applied. The results showed significant main effects of Display Type and Context but no significant Display Type × Context interaction: Display Type, *F*(1, 19) = 16.834, *p* = 0.001, *η*_p_^2^ = 0.470, mean RTs of 979 and 1,081 ms for the original and reverse displays, respectively; Context, *F*(1, 19) = 6.043, *p* = 0.024, *η*_p_^2^ = 0.241, the mean cueing effect was 54 ms (1,003 and 1,057 ms of the old and new displays, respectively); and the Display Type × Context interaction, *F*(1, 19) = 0.041, *p* = 0.841, *η*_p_^2^ = 0.002.

Taken together, these results suggest that rapidly presented contexts were acquired even when they were task-irrelevant and contained their own irrelevant target. As shown above, the acquired old yet irrelevant context from the learning session, immediately manifested the contextual cueing effect in the transfer session under longer presentation time. More importantly, we observed a comparable magnitude of contextual cueing effects before and after reversing the instructions. This finding suggests that not only the context with only the target-relevant color but also the context with a different color (in previous studies termed as “ignored” or “task-irrelevant” context) could be learned when the displays were presented under only 300 ms display presentation.

#### Recognition Task

We examined participants’ recognition performance utilizing the recognition sensitivity *d*’ [*d*’ = *Z* (hit rate) − *Z* (false-alarm rate); Green and Swets, 1966]. A hit means that participants correctly judged a “repeated” configuration as “old,” while a false alarm means that participants incorrectly judged a “novel,” random configuration as “old.” The hit and false alarm rates were 63 and 58%, respectively. The mean *d*’ was 0.11 (*SE* = 0.12) and not significantly different from zero, *t*(19) = 0.922, *p* = 0.368, Cohen’s *d* = 0.206, *BF*_10_ = 0.339, indicating that participants did not have explicit memory for old contexts.

#### Interim Discussion

Experiment 1 tested whether a rapid presentation of search displays (300 ms) would force the acquisition of task-irrelevant invariant contexts presented in a learning session. The success of such acquisition was assessed in the transfer phase, where the instructions regarding which color to attend (task-relevant and -irrelevant colors) were reversed. As a result, we observed reliable contextual cueing transfer effects for the search items that were originally presented in both task-relevant and -irrelevant colors, indicating that rapid presentation of search items induced learning of task-irrelevant invariant subsets. This finding contrasts previous results from studies with relatively unlimited presentation duration, where no contextual cueing was observed in a transfer session (e.g., Jiang and Leung, 2005). What is more, the previous studies used relatively short learning phases with 20–30 blocks (Jiang and Leung, 2005) relative to the current version with 50 blocks. Therefore, we administered Experiment 2 as a control experiment, identical to Experiment 1, except for a longer presentation (2,500 ms) of the search display in the learning phase and a higher number of learning blocks (compared to previous studies, see logic below).

## Experiment 2

To reiterate, the purpose of Experiment 2 was to examine whether the lack of learning for the target-irrelevant context in previous studies was due to the long presentation duration of the search display or purely on account of less practice. The current experiment applied a longer learning phase (50 blocks) under relatively longer presentation durations (2,500 ms). Should we observe results similar to that of previous studies (i.e., the target-irrelevant context was not acquired; Jiang and Leung, 2005) with a prolonged learning phase, we could reasonably attribute the result of Experiment 1 to the rapid presentation duration; otherwise, if we observe a transfer of contextual cueing from the target-irrelevant context, we can claim that cueing was not learned because of insufficient training.

### Method

A new sample of 20 naive participants (17 females; age range: 19–24 years) took part in the experiment. The experimental paradigm, design, and all other parameters were identical to Experiment 1 except that the displays were presented for 2,500 ms in both the learning and the transfer sessions.

### Results

#### Errors

**Figure 4:**
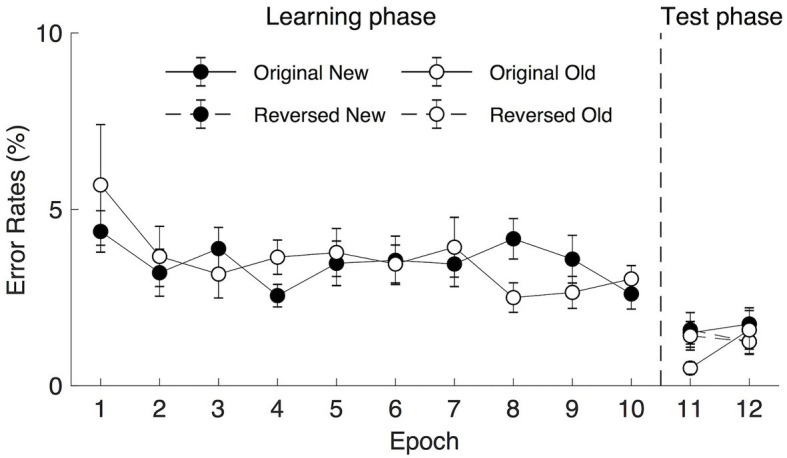
Mean error rates in the learning (epoch 1–10) and the transfer (epoch 11–12) phases as a function of epoch, context, and display type under long (2,500 ms) search display presentation. The error bars represent the within-subject SE of the mean. The black circle lines indicate the new context and the open circle lines indicate the old context. The solid line indicates the original context and the dashed line represents the reversed context.

Trials with error or no response were considered as error trials. The mean error rates were low both in the learning (2.42%, contained 0.20% miss rates) and the transfer sessions (2.71%, contained 0.10% miss rates). In the learning session, a repeated measure ANOVA on the mean error rates with Context (old and new) and Epoch (1–10) as factors failed to reveal significant main effects of Context [*F*(1, 19) = 0.363, *p* = 0.554, *η*_p_^2^ = 0.019, *BF*_10_ = 0.136, the mean error rates for the old and new contexts were 2.342 and 2.492%, respectively] and Epoch [*F*(17.519, 13.68) = 1.272, *p* = 0.289, *η*_p_^2^ = 0.063, *BF*_10_ = 0.054 (see Figure 4)]. The interaction of Context × Epoch was also not significant, *F*(9, 171) = 1.236, *p* = 0.276, *η*_p_^2^ = 0.061. In the following transfer session, a three-way repeated measure ANOVA on the mean error rates with Context (old and new), Epoch (1–2), and Display Type (reverse and origin) as factors did not reveal any significant main effect (see Figure 4): Context, *F*(1, 19) = 1.825, *p* = 0.193, *η*_p_^2^ = 0.088, *BF*_10_ = 0.464, with the mean error rates of 1.188 and 1.522% for the old and new contexts, respectively; Epoch, *F*(1, 19) = 1.234, *p* = 0.280, *η*_p_^2^ = 0.061, *BF*_10_ = 0.267; Display Type, *F*(1, 19) = 0.030, *p* = 0.864, *η*_p_^2^ = 0.002, *BF*_10_ = 0.173. No interactions were statistically significant (all *F*s < 2.192, all *p*s > 0.155). Altogether, these results suggest that error rates were comparable between contexts both in the training and the transfer phase. The error trials were excluded from further analysis.

#### Reaction Times

##### Learning Phase

**Figure 5:**
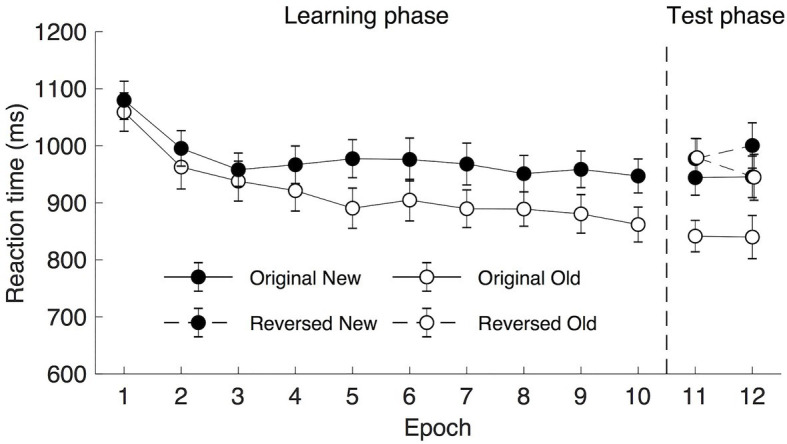
Mean RT as a function of epoch and context for long display presentation (2,500 ms). The error bars represent the within-subject SE of the mean. The black circle lines indicate the new context and the open circle lines indicate the old context. The solid line indicates the original context and the dashed line represents the reversed context, respectively.

The mean RTs are shown in Figure 5 (Epoch 1–10). A repeated measure ANOVA of RTs in the learning phase with factors Context (old and new) and Epoch (1–10) showed a significant main effect of both Context [*F*(1, 19) = 15.192, *p* = 0.001, *η*_p_^2^ = 0.444, mean RT of 919 and 977 ms for old and new contexts, respectively, mean contextual cueing = 58.056] and Epoch [*F*(4.404, 83.685) = 16.732, *p* < 0.001, *η*_p_^2^ = 0.468, mean RT reduced from 1,069 ms in Epoch 1 to 904 ms in Epoch 10]. The interaction between Context and Epoch was significant, *F*(9, 171) = 4.335, *p* < 0.001, *η*_p_^2^ = 0.186. *Post hoc* analysis showed that there was a significant difference between old and new contexts from Epoch 4 onward (all *t*s > 2.935; all *p*s < 0.009, Cohen’s *d*s > 0.656, mean contextual cueing > 45 ms), suggesting that contextual cueing effect developed with the practice of the experiment.

##### Transfer Phase

Similar to Experiment 1, a repeated measure ANOVA with factors Display Type (reverse vs. origin display), Context (old vs. new), and Epoch (11–12) was applied: For the Epoch, mean RTs were comparable between Epoch 11 and 12, *F*(1, 19) = 0.029, *p* = 0.865, *η*_p_^2^ = 0.002, *BF*_10_ = 0.177, mean of 936 and 933 ms, respectively (see Figure 5). For the Context, *F*(1, 19) = 14.945, *p* = 0.001, *η*_p_^2^ = 0.440, participants were faster in responding to the old displays than to the new displays (mean of 901 and 967 ms, respectively, mean contextual cueing of 50.47 and 81.21 ms in Epoch 11 and 12, respectively), confirming the existence of contextual cueing effect. For the Display Type, consistent with the results in Experiment 1, mean RTs were longer on the reversed displays (976 ms) than that of the original displays (893 ms), *F*(1, 19) = 23.742, *p* < 0.001, *η*_p_^2^ = 0.555. Interestingly, both the Display Type × Context [*F*(1, 19) = 8.185, *p* = 0.010, *η*_p_^2^ = 0.301] and Context × Epoch [*F*(1, 19) = 4.824, *p* = 0.041, *η*_p_^2^ = 0.202] interactions were significant, while all the other interactions did not reach significance (all *F*s < 2.034, all *p*s > 0.05), including the three-way interaction [*F*(1, 19) = 2.034, *p* = 0.170, *η*_p_^2^ = 0.097, *BF*_10_ = 0.348]. The Display Type × Context interaction was mainly caused by larger contextual cueing effect for the original display type (102.5 ms) relative to the reverse type (−1.8 ms) in Epoch 11, *t*(19) = 3.381, *p* = 0.003, Cohen’s *d* = 0.756, *BF*_10_ = 13.637, but this difference was no longer significant in Epoch 12 (original: 105.6 ms vs. reverse: 55.6 ms), *t*(19) = 1.698, *p* = 0.106, Cohen’s *d* = 0.380, *BF*_10_ = 0.785.

The Context × Epoch interaction was mainly caused by stronger contextual cueing effect in Epoch 12 (81.21 ms) relative to Epoch 11 (50.47 ms), *t*(19) = 2.256, *p* = 0.036, Cohen’s *d* = 0.504, suggesting a continuous development of contextual cueing effect in the transfer session. It is important to mention that the contextual cueing developing pattern for the reverse and original displays were different. To be specific, for the original display, only the main effect of Context [*F*(1, 19) = 25.084, *p* < 0.001, *η*_p_^2^ = 0.569, mean cueing of 104 ms] but neither the main effect of Epoch [*F*(1, 19) < 0.0001, *p* = 0.995, *η*_p_^2^ < 0.001, *BF*_10_ = 0.242] nor the interaction [*F*(1, 19) = 0.017, *p* = 0.898, *η*_p_^2^ = 0.001] were significant, suggesting comparable magnitudes of contextual cueing effect in the two transfer epochs. By contrast, for the reversed displays, a repeated measure ANOVA with Context and Epoch as factors revealed a significant Context × Epoch interaction [*F*(1, 19) = 6.557, *p* = 0.019, *η*_p_^2^ = 0.257], but no significant main effects [Context, *F*(1, 19) = 1.433, *p* = 0.246, *η*_p_^2^ = 0.070, *BF*_10_ = 0.624; Epoch, *F*(1, 19) = 0.116, *p* = 0.738, *η*_p_^2^ = 0.006, *BF*_10_ = 0.240]. The interaction was mainly caused by no contextual cueing effect in the first transfer epoch [Epoch 11, *t*(19) = 0.069, *p* = 0.945, Cohen’s *d* = 0.016, *BF*_10_ = 0.233, mean RTs were 979 and 977 ms for the old and new displays, respectively], but a significant contextual cueing effect in the second transfer epoch [Epoch 12, *t*(19) = 2.335, *p* = 0.031, Cohen’s *d* = 0.522, mean RTs were 945 ms and 1,001 ms for the old and new displays, respectively, mean contextual cueing = 56 ms]. Note that the contextual cueing effect observed in the second epoch of the transfer session was comparable to the effect of 45 ms in Epoch 4, *t*(19) = 0.412, *p* = 0.685, Cohen’s *d* = 0.092, *BF*_10_ = 0.251. This suggests that new learning of the reversed displays was possible, and the learning speed was even faster than that of the initial learning speed. Restate, participants took four epochs to obtain significant contextual cueing effect in the initial learning phase, while only two epochs were required in the transfer phase. To summarize, with longer presentation duration (2.5 s), the task-irrelevant context was not acquired in the learning session, hence no contextual cueing transfer effect was observed in the first transfer epoch. However, with one epoch (five blocks) of repetition, participants were able to build the contextual relationship between the target with the previously irrelevant color and its respective distractors.

#### Recognition Task

Both the hit and false alarm rates were ~58%. The mean recognition sensitivity *d*’ was 0.03 (*SE* = 0.13) and not significantly different from zero, *t*(19) = 0.202, *p* = 0.842, Cohen’s *d* = 0.045, *BF*_10_ = 0.237, indicating that participants did not have explicit memory for old contexts.

#### Interim Discussion

The purpose of Experiment 2 was to account for potential confounding factors and inconsistencies between the rapid presentation Experiment 1 and previous studies that tested the role of task-relevance in the acquisition of context regularities. To this end, participants had more blocks and longer trials during both the learning and the transfer phase in Experiment 2. As a result, Experiment 2 could replicate the major findings of previous works. Specifically, we showed that contextual cueing could develop in the learning phase of the study under a longer presentation duration. Most importantly, in the transfer phase, when participants were instructed to search for the target of a different color with the whole display keeping their original features, the cueing effect was only pronounced for those invariant subsets that were task-relevant in the learning phase. Interestingly, the lack of contextual facilitation for the task-irrelevant context lasted for only one epoch (in Epoch 11). With more blocks of repetition, participants could learn the associations between the original task-irrelevant context and show significant contextual facilitation already in Epoch 12. This observation suggests that the associative blocking is not absolute, and may not last for a long time. The meaning and implications of both Experiments will be further discussed in the general discussion section below.

## General Discussion

The current work set out to investigate the nature of associative blocking in the contextual cueing task. Previous studies showed that invariant configurations of items presented in *task-irrelevant* color and together with a different subset of invariant items in *task-relevant* color (i.e., both-old conditions) did not facilitate visual search in the transfer phase when instructions regarding task-relevant and -irrelevant subsets were reversed (Jiang and Leung, 2005). In contrast, when the invariant task-irrelevant context was paired with novel task-relevant context during the training phase (i.e., ignored-old condition), there was no cueing effect in the initial learning phase, but the search performance was facilitated immediately after the instructions regarding task-relevant-irrelevant subsets was reversed in the transfer phase (Jiang and Leung, 2005). This suggested that the task-irrelevant old context was learned during the early training session in the ignored-old condition, but not in the both-old condition. The phenomenon was explained in terms of associative blocking mechanisms where salient relevant cues (invariant displays in task-relevant colors) blocked the learning of the association between less salient cues and target (invariant displays in task-irrelevant colors), thus hindering the acquisition (learning) of such associations. An alternative account would be that associative blocking hinders the attentional selection of task-irrelevant items, which hinders learning. The current work used rapid presentation design to contrast these two alternatives. It tested whether forcing participants’ attention to both task-relevant and -irrelevant invariant subsets of items would improve the post-transfer performance of these configurations.

In two experiments, participants performed the contextual cueing search task under rapid (300 ms; Experiment 1) or standard display presentation (2,500 ms; Experiment 2) in a learning session where they had to search for a predefined colored target (i.e., black or white) among white and black distractors. For a successful task performance in this phase, it was enough to concentrate on the task-relevant subset of items, while ignoring the other group of items that never contained the target item and were thus irrelevant (i.e., task-irrelevant contexts). In a transfer session, the participants performed the same task, but unbeknownst to them, for half of the configurations, the instructions regarding the relevance of context were reversed. For example, a context previously irrelevant (relevant) in the learning session became relevant (irrelevant) in the transfer session. For the other half of the configurations, the instructions remained the same as in the learning session to serve as the baseline. We demonstrated that the learned contextual cueing effects under fast display presentation could be transferred to displays with reversed instructions. More importantly, we observed comparable contextual cueing effects for both original and reversed instruction displays, suggesting that not only the task-relevant context but also the task-irrelevant context was learned when the displays were presented for only 300 ms in the initial training session. In Experiment 2, where the displays were presented for 2,500 ms, the contextual cueing was also reliable in the learning session. Crucially and in contrast to Experiment 1, the task-irrelevant context was not learned in Experiment 2, as there was no transfer of contextual cueing when the instructions regarding the relevance of context were reversed.

The finding that rapid display presentations of only 300 ms facilitate the acquisition of task-irrelevant context provides new evidence for the importance of the role of selective attention in contextual learning, which could potentially link selective attention and the associative blocking mechanism. Specifically, the 300 ms presentation might be too fast to allow the participant to control the overt distribution of attentional allocation across the entire visual display. That is, to ignore part of the display (i.e., irrelevant stimuli), it is first necessary to have at least a glimpse of the global space to determine which visual items are to be attended vs. ignored. As a result, under fast display presentation, participants form a global context representation of the configuration of items (see also Navon, 1977; Gerlach and Poirel, 2018 for the global precedence account), and inevitably attend to both task-relevant and -irrelevant stimuli, thus combining both types of contexts into one contextual memory. In addition, the results obtained from the analysis of error responses were also in accordance with this idea. For instance, the error rate was relatively high when the display presentation duration was short (300 ms in Experiment 1) compared with a longer presentation time (2,500 ms in Experiment 2). The high error rate observed under short display presentations could be interpreted as the failure to process the display’s minor details by limiting selective attention and local processing. That is, the short 300 ms presentation time may only be enough for coarse processing, such as global, nonselective processing (Fabre-Thorpe, 2011; Wolfe et al., 2011), which in turn brings about higher sensory noise. This high sensory noise, together with a potential high decision-making noise induced by the response limit, would finally generate more error responses. To summarize, despite some obvious differences between the experiments, such as the global RT and error rate differences, the current results jointly speak in favor of the role of selective attention in associative blocking.

The idea that the global processing may cause the learning of task-irrelevant context under rapid presentation is also supported by a previous rapid presentation contextual cueing study (Xie et al., 2020). It revealed that the repeated local context only within a single quadrant containing the target, which was proved to be sufficient to generate contextual cueing effect with a long display presentation time (i.e., 8 s) in the study of Brady and Chun (2007), could hardly be acquired under the 300 ms rapid presentation condition (Xie et al., 2020). This suggests that global context should stay invariant to guide visual search under rapid presentation. Applied to the current work, the learning of the task-irrelevant context could not be blocked under fast presentation because attention was directed to both the task-relevant and -irrelevant contexts. Moreover, these results are also with the suggestion by Vadillo et al. (2020) that previous studies underestimated the importance of selective attention in contextual cueing (Jiang and Chun, 2001; Jiang and Leung, 2005). For instance, it demonstrates that attentional selection is important for contextual learning; however, task requirements determine which level of perceptual learning can be achieved. This can be illustrated with the longer exposure time in Experiment 2, in which the segregation into fore/background may naturally take place after global processing (see also Zang et al., 2016). This is probably because participants have sufficient time to zoom in from the global level to the highly detailed information processing, such as the processing of the task-relevant search items, thus resulting in the display segregation and the local focus of the attention, and in turn building a spatial representation of the task-relevant but not the task-irrelevant context in contextual memory.

It is interesting to note that, with longer display presentations, the global spatial representation of the display that contains association among the target and all (both task-relevant and irrelevant) items may be held only temporarily in memory, until the participants correctly segregate the display. To be specific, global representation is no longer sustained when attention is correctly directed to the most informative, salient task-relevant context, thus freeing up cognitive resources for further processes. This selective attention or the “attentional control” system helps the human to optimally allocate the limited cognitive resources to the relevant information (Bundesen, 1990; Hakim et al., 2021). Thus, it is plausible to claim that further cognitive processes such as display segregation and attentional allocation block the associations between the target and the ignored distractors through either overwriting the global representation or making the global information of the current display obsolete, thus extinguishing it from contextual memory since it is no longer needed for completing the task. In support of this claim, with long display presentations (10 s), Brady and Chun (2007) proposed that spatial contextual cueing could be constructed purely on the local context that appears in the target’s quadrant, whereas Olson and Chun (2002) proposed that long-distance context that is far away from the target did not facilitate contextual learning. Together with our findings, these studies demonstrate that the contextual cueing effect may take different perceptual routes to manifest, which is highly dependent on the task requirements that ultimately dictate the levels of processes necessary to achieve the effect.

It is worth mentioning that in contrast to previous studies that tested the potential transfer of the cueing effect with only two blocks of trials in the transfer phase (e.g., Jiang and Leung, 2005), the current work used a rather extensive transfer session including 10 blocks. This was done to explore whether, under long presentation conditions over several blocks, associative blocking would remain as a persistent phenomenon that blocks the new learning of the previously ignored invariant contexts even when they become the task-relevant context in the transfer session. Interestingly, the associative blocking disappeared already in the second Epoch of the transfer phase (i.e., Epoch 12 of Experiment 2) as we found a reliable cueing effect in this Epoch. This finding suggests that associative blocking does not tag “blocked” contexts as contexts that should not be learned in the future. Instead, it blocks non-informative context only when it can implicate the performance of the task at hand. In other words, participants may perceive the display types in the transfer session as “fresh” contexts, thus going through the processes required for the contextual cueing task anew for the reversed displays. Once those previously blocked task-irrelevant contexts become task-relevant, they are no longer blocked, they can be learned and, therefore, generate the contextual cueing effect without any interference from the previously relevant context. This secondary finding is a great avenue for future research as it adds new information to the idea that the cueing effect was likely not acquired during the initial learning due to lack of selective attention, rather than due to blocking of learning.

Interestingly, it seems that context learning in the transfer phase was acquired faster (epoch 2) relative to that in the learning phase (epoch 4). This could be potentially explained by the proposed “learning-to-learn” account (Bavelier et al., 2012), which suggested that participants learned to acquire new information in the initial learning phase, and these learning skills could be naturally used to advance performance later during the task. For instance, Zhang et al. (2020) reported that learning to play action video games improved participants’ learning rate in an N-back working memory learning task. In the same vein, we showed that the learning of the original search display in the initial learning session increased participants’ learning ability as well as their “expectations” about the repetition of the environmental regularities (i.e., repeated displays) in the visual search task. As a result, an increased learning rate of the contextual cueing effect was observed, even though the “reversed” displays may have been seen as completely “fresh” displays in the transfer session. This finding is in line with the work by Jungé et al. (2007). Those authors presented participants with an initial block of 100% repeated or 100% non-repeated displays, followed by the performance of the usual contextual cueing task where only 50% of displays were repeated. It was shown that only those participants who started with 100% repeated displays, showed contextual benefits in the subsequent task, suggesting that the contextual cueing effect was stronger when participants learned and obtained certain expectations/beliefs about the existence of statistical regularities (i.e., repeated displays). A recent study found that global repetition frequency formed by different presentation ratios between the repeated and non-repeated configurations influences contextual cueing effect (Zang et al., 2018; Zinchenko et al., 2018), further confirming that global expectations can further modulate contextual memories. Applied to the current findings, it is reasonable that once participants form some expectations about the environmental regularities (i.e., about the existence of repeated displays), it is easier to acquire new contextual associations.

Regarding the transfer session with reversed instructions, one could argue that even though participants were told to search for a previously ignored target, they may still attend to the entire context. That is, even if they had to attend to a given color in the transfer session (unattended items in the learning session), the current unattended color (items attended in the learning session) still provided contextual information about the target. Nevertheless, we can confidently dismiss this alternative since both attended and unattended items contained a “T”-shape item that served as the target within that group. That is, two different targets, one black and one white, were presented in each display [which is different from Jiang and Leung (2005) study that contained only one target in each display]. If anything, instead of boosting search, irrelevant stimuli would be more likely to slow down search performance since it would first guide search to its own irrelevant target. Nevertheless, this is a question worth investigating in the future; since each color group had its own target, it would be interesting to test whether the unattended group of items would improve the target search of the attended target (but now in the same color). This would demonstrate whether all items are learned as one display regardless of distinctive sets (under 300 ms), or if the global representation is subliminally segregated, in other words, irrelevant items only improve the search of its own color-defined target. Back to the potential attendance of the entire display in the transfer session, if it was true, we should have observed task-irrelevant context being learned when the displays were presented for a longer duration in Experiment 2.

To summarize, the current study demonstrated that the contextual cueing effect is supported by perceptual processes that are modulated by task requirements. For example, given sufficient time exposure, different levels of processing (i.e., global processing, segmentation, and local processing) can take place to perform the task as close as possible to its requirements. However, such a thorough process does not allow for the storage of task-irrelevant information in memory, supporting the associative blocking idea; more precisely, the blocking of attention. Nevertheless, under certain conditions such as rapid exposure, global precedence alone may dictate the outcome of the task, as further levels of perceptual processing do not take place. Thus, global representation including both task-relevant and irrelevant contexts may be represented in memory, leading both to be learned in contextual memory.

## Data Availability Statement

The original contributions presented in the study are included in the article/supplementary material, further inquiries can be directed to the corresponding authors.

## Ethics Statement

The studies involving human participants were reviewed and approved by the Ethics Committee of the Institute of Psychological Sciences in Hangzhou Normal University. The patients/participants provided their written informed consent to participate in this study.

## Author Contributions

XZ contributed to the study design, data analysis, and manuscript composition. LA engaged in data interpretation and manuscript composition. JW made contributions to data analysis and manuscript composition. XX contributed to data collection, data analysis, and preparation of the manuscript. AZ mainly engaged in data interpretation and manuscript composition and preparation. All authors contributed to the article and approved the submitted version.

### Conflict of Interest

The authors declare that the research was conducted in the absence of any commercial or financial relationships that could be construed as a potential conflict of interest.
